# A single-center experience in the use of hybrid techniques for thoracic aortic pathology

**DOI:** 10.25122/jml-2021-0284

**Published:** 2022-02

**Authors:** Igor Oleksandrovych Ditkivskyy, Vitaly Ivanovich Kravchenko, Oleksandra Oleksandrivna Lohvinenko, Michael Ivanovich Sheremet

**Affiliations:** 1. Department of Interventional Cardiology for Congenital and Acquired Heart Disease, Amosov National Institute of Cardiovascular Surgery of National Academy of Medical Sciences of Ukraine, Kyiv, Ukraine; 2. Department of Surgical Treatment of Aortic Pathology, Amosov National Institute of Cardiovascular Surgery of National Academy of Medical Sciences of Ukraine, Kyiv, Ukraine; 3. Surgery Department No.1, Bukovinian State Medical University, Chernivtsi, Ukraine

**Keywords:** aortic aneurysm, aortic dissection, hybrid treatment, endovascular prosthesis of the thoracic aorta, debranching, carotid-subclavian bypasses, TEVAR – thoracic endovascular aortic repair, PAU – penetrating aortic ulcer, TAAD – type A aortic dissection

## Abstract

The hybrid method of treating thoracic aortic pathology is devoid of the disadvantages of traditional open surgery and, at the same time, has a broader range of applications than the endovascular method. From 2014 to 2019, we researched 122 patients with thoracic aortic pathology treated with the hybrid method (open surgery with thoracic endovascular aortic repair (TEVAR) at the National M. Amosov Institute of Cardiovascular Surgery National Academy of Medical Science of Ukraine. In the general group, 34 patients had a descending aortic aneurysm without dissection or rupture; 71 patients had an aortic dissection (10 – acute, 9 – subacute, 52 – chronic), penetrating aortic ulcer (PAU) – 7, thoracoabdominal aneurysm (Crawford I-II) – 4, isolated aortic arch aneurysm – 2, residual enlargement aorta after previous ascending aortic grafting causing type A acute aortic dissection (TAAD) – 3, primary aortic thrombosis – 1. Patients admitted as elective surgery candidates had switched aortic arch vessels (debranching) in the first stage and TEVAR in the second stage. For emergencies (aortic rupture with uncontrolled bleeding, malperfusion syndrome), TEVAR operation was performed first. Long-term results of treatment after three and six months are satisfactory. The hybrid technique of aortic arch treatment using modern minimally invasive technologies can eliminate the pathology in the most dangerous segment of the main artery of the body, providing a reasonable level of hospital mortality – 4.2%, and a small number of neurological complications.

## Introduction

Nowadays, open surgery is considered the gold standard for treating ascending aorta and aortic arch pathology. However, traditional surgical methods to correct aortic arch pathology are invasive and are often associated with a significant systemic inflammatory response syndrome and complications. Therefore, patients with multiple comorbidities are often classified as high-risk and are denied open aortic surgery and aortic arch surgery, given the prevalence of lesions in ischemic zone 2. For the first time, the idea of the minimally invasive, endovascular method of aortic aneurysm repair was proposed by M.L. Volodos [1, 2]. For various reasons, the method was developed and widely used in Western European medicine, and only in the last few years has it become widely used in Ukrainian cardiac surgery, particularly in our facility. The advantage of endovascular and hybrid techniques, compared to the open method of care, is to reduce perioperative complications and duration of treatment. The severe postoperative period following a standard surgical option, such as complete replacement of the aortic arch or the use of a hybrid technique of stabilized/frozen elephant trunk, show a fairly high incidence of hospital mortality and cerebrovascular disorders up to 9% and 4–12% respectively [3, 4–6, 7].

On the other hand, minimally invasive treatment of aortic arch pathology faces some technical problems. First, brachiocephalic vessels perfuse the brain, which has low ischemic tolerance. In addition, the aortic arch is a wide, angular, pulsating structure and is far from typical access vessels, such as the femoral arteries. Also, the presence of significant atheromatous masses and thrombus in the aortic arch (*i.e.*, shaggy aorta) increases the risk of developing cerebral embolism [8–9]. Finally, it is necessary to remember the critical role of maintaining left subclavian artery (LSA) blood flow to prevent spinal ischemia [10].

The endovascular and hybrid approach to treating aneurysms of the arch and descending thoracic aorta and their dissections makes it possible to provide care even to high-risk patients. However, the anatomy of the thoracic aorta should be favorable for the establishment of the endograft. Complex forms of thoracic aortic aneurysms and dissections extending to the aortic arch can thus limit the possibilities of conventional endovascular care. Until now, the long-term results of the endovascular approach have not been definitively studied and systematized. Conventional surgical replacement of the aortic arch in the conditions of artificial circulation remains the optimal treatment method for patients at low risk. The advantage of the hybrid or isolated endovascular method can be demonstrated in complex cases complicated by numerous concomitant conditions in the older age group of patients. This approach involves the translocation of large brachiocephalic branches combined with endograft stent implantation [11–14]. This study aimed to present a facility experience in the hybrid treatment of complex pathology of the thoracic aorta.

## Material and Methods

From 2014 to 2019, 122 patients with aortic pathology underwent hybrid treatment at the Amosov National Institute of Cardiovascular Surgery (ANICS): switching of branches of the aortic arch (debranching) and endovascular prosthesis of the descending aorta by stent-graft (TEVAR). The group was dominated by men (n=86). The age of patients ranged from 22 to 91 years, the mean age 57.8±11.2 years. The classification of patients by diagnosis is given in Table 1.

**Table 1.** Classification of patients based on aortic aneurysm formation.

**Diagnosis**	**Number of patients**	**%**
**Thoracic aortic aneurysm (TAA)**	34	27.9%
**Post-traumatic descending aorta aneurysm**	9	7.4%
**Post-coarctation descending aorta aneurysm**	18	14.8%
**Thoracic aortic aneurysm of unknown etiology**	7	5.7%
**Aortic dissection**	71	58.2%
**Acute**	10	8.1%
**Subacute**	9	7.3%
**Chronic**	52	42.6%
**Penetrating aortic ulcer**	7	5.7%
**Thoracoabdominal aneurysm (Crawford I-II)**	4	3.3%
**Isolated aortic arch aneurysm**	2	1.6%
**Aortic dissection type A (ascending aortic prosthesis)**	3	2.5%
**Primary aortic thrombosis**	1	0.8%
**Total**	122	100.0%
**Including hemothorax**	7	5.7%

Approximately 1/3 of patients (n=34) had a thoracic aortic aneurysm without dissection or rupture. According to the etiology, the most common were post-coarctation and post-traumatic aortic aneurysms (n=18; n=9, respectively). Many patients (n=71) had an aortic dissection. Chronic aortic dissection of type A according to the Stanford classification was observed in 52 patients. Three patients had previously undergone ascending aortic prosthesis (in one case – Bentall de Bono surgery 19 years before the current hospitalization; in two cases – supracoronary ascending aorta replacement for a year and 4 years before the current hospitalization); the fourth patient did not undergo surgical treatment before hospitalization in ANICS. Type B aortic dissection was observed (n=9 chronic; n=2 subacute; n=4 acute) in the remaining patients. In 7 patients, aortic dissection was complicated by left-sided hemothorax; in three – by malperfusion syndrome; in one – by hemothorax and malperfusion syndrome. Also, two patients had a ruptured aorta without dissection, accompanied by left hemothorax; one patient was diagnosed with a penetrating aortic ulcer.

For patients admitted with scheduled surgery or without life-threatening conditions, the branches of the aortic arch were switched, followed by endovascular prosthesis of the descending aorta. Patients with life-threatening conditions (malperfusion syndrome, rupture of the aorta with hemothorax) got the TEVAR procedure first, and after the patient was stabilized, switching of the vessels of the aortic arch was performed. Also, in the presence of hemothorax, patients underwent one-stage drainage of the pleural cavity intraoperatively after stent-graft implantation. If necessary, depending on the anatomical features, the LSA ostium was occluded (if there was endoleak from LSA).

Table 2 shows the structure of the open operations performed. Switching of vessels of an aortic arch (debranching) depending on the quantity of the vessels reimplanted in an ascending aorta is divided into partial debranching – with cutting off and reimplantation of one vessel; subtotal – with reimplantation of two vessels; total – with cutting and reimplantation of all branches of the aortic arch [8]. Patients with ascending aortic lesions were simultaneously treated for ascending aortic lesions (complete debranching with supracoronary ascending aortic replacement, complete debranching with the Wheat procedure, and Conventional Elephant Trunk (CET), complete debranching with CET. In addition, a patient with a coarctation-associated descending aortic aneurysm and concomitant aortic stenosis underwent Robicsek procedure two weeks after hybrid treatment of the descending aorta.

**Table 2.** Classification of open operations performed.

**Procedure**	**Number of patients**	**%**
**Partial debranching**	50	56.8%
**Subtotal debranching**	26	29.5%
**Total debranching**	6	6.8%
**Total debranching and supracoronary prosthesis of the ascending aorta**	2	2.3%
**Total debranching, Wheat procedure, and CET**	1	1.1%
**Full debranching and CET**	1	1.1%
**Visceral debranching**	2	2.3%
**Total**	84	100.0%

In the remote period, patients were monitored at 3 and 6 months after surgery and each year at follow-up. After 3 months, ultrasound examination was performed, and in case of complaints – CT aortography; after 6 months, all patients underwent CT aortography.

## Results

Mortality within 30 days was 3.3%. 3 patients died during the early postoperative period due to ascending aortic thrombosis and renal failure, acute cardiovascular insufficiency, and myocardial infarction on the 2^nd^ day after surgery. Another patient died on the 5^th^ day; the patient was transferred to another facility after the fourth day following the operation and died suddenly the next day.

The mortality rate in the three months was 4.1%. One patient died 3 months after successful aortic dissection type B surgery during the acute phase. At the discharge, retrograde aortic dissection was the cause of death. There were no deaths at the follow-up stage three to six months after treatment.

Endoleak type I was observed in 11 patients during the procedure. In seven patients, the endoleak was negligible; in four – significant, so in the latter case, patients underwent X-ray endovascular dilatation (ED) of the proximal part of the stent. Six months later, on the CT aortogram, endoleak type I was observed in one patient; the patient underwent endovascular closure with Nit-occlude Lê VSD. Type II endoleak was observed in 8 cases after stent-graft placement, and it was minimal in two cases. In six cases, the endoleak source (left subclavian artery; in one case – the brachiocephalic trunk) was closed by an occlusion. No endoleak type II was detected on CT aortograms after three and six months. In one patient with chronic type B aortic dissection after partial debranching during angiography, insufficient filling of the left renal artery from the false lumen was observed, and no visible fenestration was detected. To preserve the blood supply to the left kidney after implantation of the aortic stent graft, intima perforation was performed between the true and false lumens at the level of the renal arteries and ED fenestration of the false canal. In a patient with a chronic type B aortic dissection, TEVAR was performed two months after subtotal debranching due to technical features (prolonged waiting for a stent-graft). When performing TEVAR, thrombosis of the carotid-subclavian anastomosis was detected. On the same day after endovascular intervention, the patient underwent re-carotid-subclavian bypass. In one case, after implantation, the stent-graft took the wrong position along the internal curvature of the aortic arch with the configuration of a “bird’s beak”. After tightening the stent-graft loop from the right subclavian artery, the position of the stent was satisfactory. In a patient with chronic type B aortic dissection, after bilateral carotid-subclavian bypass in the first stage of treatment, after performing TEVAR and closing the subclavian arteries with occludes, the left vertebral artery was blocked, and during occlude removal, the left subclavian artery dissection appeared. The same patient had hoarseness due to trauma of the recurrent laryngeal nerve. Complications of treatment are shown in Table 3.

**Table 3.** Complications of the hybrid method of treatment of thoracic aortic pathology.

**Complications**	**Number, (%)**
**Endoleak type I in the remote period**	1 (0.8%)
**Thrombosis of the carotid-subclavian anastomosis**	1 (0.8%)
**Left subclavian artery dissection**	1 (0.8%)
**Recurrent laryngeal nerve injury**	1 (0.8%)
**Exitus letalis up to 3 months after surgery**	5 (4.1%)
**Total**	9 (7.4%)

## Discussion

The hybrid approach of treating aortic arch aneurysms consists of pre-made shunts from the ascending aorta to the brachiocephalic arteries – a technique of total switching of all vessels of the aortic arch – total arterial debranching; or switching of the left carotid and left subclavian artery from the brachiocephalic trunk – subtotal debranching; and/or carotid-subclavian bypass – the method of partial debranching. This technology has shown promising results over the past 10 years and has expanded the treatment of aortic arch pathology in patients considered unsuitable for open surgery [11, 12, 15]. Data from colleagues show that treatment of aortic pathology is still associated with 13% 30-day mortality, and the risk of complications in treating such pathology can reach 35% [16–18]. Damage to the aortic arch in Ishimaru zones 0 and 1 carries significantly increased risks compared to patients in whom the disease is localized distally and, accordingly, the required landing zone for endoprosthesis is located in area 2 or 3 Ishimaru [16, 19]. Hagan and Nienaber *et al.* confirmed these results and concluded that most deaths occurred due to the development of cerebral circulatory disorders in patients with stent-grafts in Ishimaru zone 0 and I [20].

Despite significant progress in developing diagnostic techniques, anesthetic, and intensive care supply, surgical treatment of aortic arch aneurysms, especially complicated by dissection, remains a high-risk procedure. The results of this type of care, even at the present stage of development of cardiac surgery, are accompanied by fairly high rates of hospital mortality – up to 25.6% (average 17.5%). An alternative to traditional surgical prosthetics of the aortic arch are hybrids: combined implantation of stent-grafts and pre-performed translocations of brachiocephalic vessels. There are reports of using a triple branched graft, and Desai *et al.* recently reported the implantation of a branched stent-graft into the aortic arch and the anonymous artery simultaneously [21, 22]. On the other hand, the medium-term results of this technique in the treatment of aortic arch aneurysms and dissections with spread to the arch and descending aorta are insufficiently studied. Numerous proposed techniques for translocation of brachiocephalic vessels even include the proposal of Criado *et al.* for retrograde shunting from the right common iliac artery to the anonymous artery [23]. However, such an alternative does not seem satisfactory in terms of vascular isolation of the brain and should be considered an exception if it is necessary to release Ishimaru zone 0 or 1, allowing a complete blood supply to the brain.

In our experience, we transplant LSA in all cases to prevent ischemic spinal cord injury. However, one should always keep in mind the role of LSA in providing coronary circulation through the left internal mammary artery, when the contralateral vertebral artery (VA) is stenosed or is in hypoplasia, and when there is an open Velizian circle with the incomplete fusion of both VAs. We also used LSA switching in association with the left carotid artery when included in the aneurysm, even though LSA is difficult to achieve with a median sternotomy, especially a J-shaped mini sternotomy. In contrast to numerous reports from foreign colleagues who switched LSA only later, it becomes symptomatic. Translocation of brachiocephalic vessels shows a small number of neurological complications. Our experience did not show any serious strokes or deaths related to translocation. After the endovascular stage, three early deaths (2.4%) were associated with complications: retrograde dissection of the ascending aorta, hemopericardium, and tamponade. Another patient, the fourth of the total, died on the 5^th^ day (the 4^th^ day after the surgery, he was transferred to another facility and died suddenly the next day). In all groups of arch vascular translocation, no negative neurological complications occurred either at the surgical or endovascular stages.

On the other hand, we observed one major stroke in a patient with an acute LSA closure, as shown by a retrospective pathological study – caused by severe hypoplasia of the contralateral vertebral artery. The risks of material embolism should also be considered in patients with thrombosed aortic arch aneurysms or “shaggy aorta” in patients with severe atherosclerotic aorta. Complete switching of the aortic arch vessels provides the longest possible landing area of endograft implantation, reaching 3 cm in length for better endograft fixation. This technique also avoids positioning the stent-graft directly in the aortic arch, which can cause endoleak development and migration. We used a step-by-step procedure for the following reasons: reduced working time, risks, and blood loss. The future of this sophisticated approach depends on whether endotransplantation technology is reliable or not. Improving stent implantation techniques is necessary for mid-aortic delivery system navigation and reducing embolic risk. Longer endografts at least 20 cm long allow the vault to cover the entire arch, thus avoiding the technique of layering one stent on another, which can lead to the development of endoleak. In our opinion, the overlap between endografts in the area of greatest curvature increases the risk of device migration and type 3 leaks. The keys to success are pre-planning the procedure to optimize the landing area and selecting the best endograft options.

## Conclusions

Aortic arch pathology is an extremely complex and difficult-to-correct condition, the surgical treatment of which is accompanied by a high percentage of hospital mortality and a high risk of neurological complications. The hybrid technique of aortic arch treatment allows using modern minimally invasive technologies to eliminate the pathology in the most dangerous segment of the main artery of the body, providing a decent level of hospital mortality – 4.2%, and a small number of neurological complications. Another advantage of the demonstrated technique is the ability to provide care to older patients with numerous concomitant complicated comorbidities.

## Acknowledgments

### Conflict of interest

The authors declare no conflict of interest.

### Ethical approval

Ethical approval for this study was obtained from the Medical Ethics Committee of National Amosov Institute of Cardiovascular Surgery of the National Academy of Medical Sciences of Ukraine, Kyiv, Ukraine (approval ID: no 1.09/21; 16.09.2021).

### Consent to participate

Written informed consent was obtained from the participants.

### Authorship

IOD contributed to the methodology and data curation. VIK contributed to data analysis and writing the original draft. OOL contributed to data collection and editing the manuscript. MIS contributed to conceptualizing the study.
